# eTEST: Developing a Smart Home HIV Testing Kit that Enables Active, Real-Time Follow-Up and Referral After Testing

**DOI:** 10.2196/mhealth.6491

**Published:** 2017-05-08

**Authors:** Tyler Wray, Philip A Chan, Erik Simpanen, Don Operario

**Affiliations:** ^1^ Department of Behavioral and Social Sciences Brown University School of Public Health Providence, RI United States; ^2^ Division of Infectious Diseases Brown University Alpert Medical School Providence, RI United States

**Keywords:** HIV, smartphone, Internet, counseling, referral and consultation, qualitative research

## Abstract

**Background:**

Men who have sex with men (MSM) are the group at highest risk for contracting human immunodeficiency virus (HIV) in the United States, but many do not test as frequently as recommended. Home-based self-testing (HBST) for HIV holds promise for promoting regular testing among these individuals, but currently available HBSTs have limited follow-up options, providing only a 1-800 number that participants can call. Failure to actively conduct follow-up counseling and referrals after HBST use could result in delays in seeking confirmatory testing and care among users receiving reactive (preliminary positive) test results. HBST also fails to connect users who test negative with other prevention services that can reduce their future risk for HIV.

**Objective:**

The aim of our study was to use qualitative research methods with high-risk MSM to inform development of a “smart” HBST kit. The kit utilizes existing Internet-of-Things (IoT) technologies to monitor HBST use in real-time and enable delivery of timely, active follow-up counseling and referrals over the phone.

**Methods:**

In phase 1, individual interviews (n=10) explored how participants might use HBST and their views and preferences for conducting counseling and referral after HBST. Based on these perspectives, we developed a smartphone app (iOS, Android) that uses data from light sensors on Bluetooth low energy (BLE) beacons to monitor when HBST kits are opened, facilitating timely follow-up phone contact with users. In phase 2, a usability study conducted among high-risk MSM (n=10) examined the acceptability and feasibility of this system and provided user perspectives after using the system along with HBST.

**Results:**

Phase 1 themes suggested that MSM preferred HBST, that most thought active follow-up after HBST would be valuable, and that doing so over the phone within 24 h after testing was preferable. Phase 2 results showed that the eTEST system successfully detected HBST use in nearly all cases. Participant perspectives also suggested that the timing, method (ie, phone call), and duration of follow-up were appropriate and helpful.

**Conclusions:**

Using BLE beacons and a smartphone app to enable follow-up counseling and referral over the phone after HBST use is feasible and acceptable to high-risk MSM. Future research is needed to compare the effects of follow-up counseling on rates of repeat testing and receipt of referral services (eg, testing for sexually transmitted infections and initiation of preexposure prophylaxis) and to explore the acceptability of the eTEST system over longer periods of time.

## Introduction

New human immunodeficiency virus (HIV) infections among men who have sex with men (MSM) in the United States continue to climb [1], with recent data showing that up to 1 in 6 MSM will be diagnosed with HIV in their lifetimes [2]. Modeling studies suggest that up to 50% of new HIV infections among MSM stem from the approximately 20% of those who are infected with HIV but are unaware of their status and thus are not virally suppressed [3-5]. These data highlight that an important step toward reducing HIV incidence in MSM involves increasing the accessibility and regularity of testing [6].

**Figure 1:**
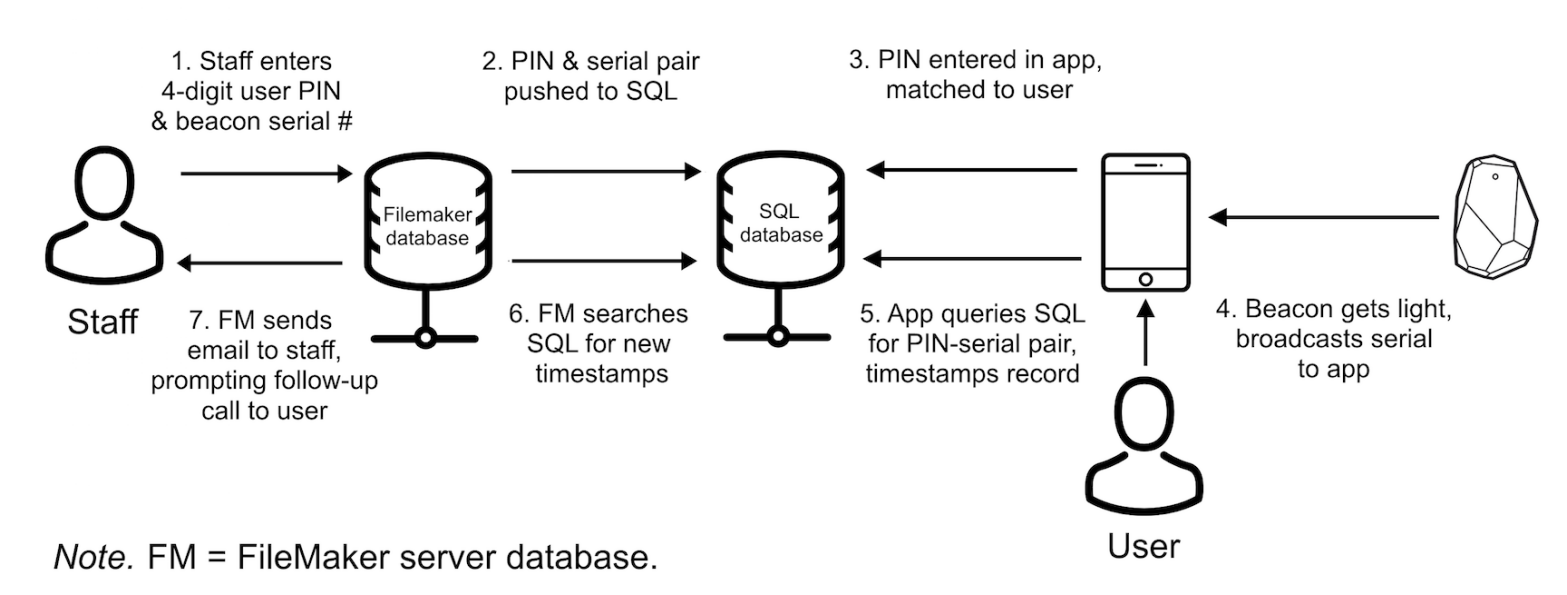
Flow of eTEST system components.

The first rapid antibody HIV test designed to be used and interpreted entirely by consumers at home was approved for over-the-counter sale by the US food and drug administration (FDA) in 2012 (OraSure Technologies, Bethlehem, PA; see Figure 1). The OraQuick home-based self-test (HBST) for HIV samples oral fluid and produces results in 20 min, and it has instructions that are simple and easy to understand. HBSTs could provide opportunities to expand and encourage regular HIV testing, especially among those who encounter barriers to testing at standard “brick-and-mortar” clinical sites. Research in developing countries using blood sample HBSTs has shown higher uptake of HBST compared with clinic-based testing [7-9]. Moreover, past studies in the United States show that many MSM, including those who have never tested, prefer to do so at home and believe they would test more often with HBST [10-15], since HBST addresses key barriers to clinic-based testing (eg, confidentiality and inconvenience [10,16]). Finally, past studies using popular gay-oriented social networking smartphone apps to connect high-risk MSM with free HBST showed that many users requested kits, and that they were useful for detecting new HIV infections among these users [17-20]. Moreover, 77% of these new cases were ultimately diagnosed at CD4 counts >350 cells/μL, suggesting that this approach may facilitate early diagnosis and treatment [20].

Despite their promise for overcoming barriers to testing, currently available HBSTs also have a number of important limitations. In some studies, the OraQuick test has shown lower sensitivity (95.9-99.6%) compared with other rapid antibody tests, especially when oral fluid is used [21,22]. The OraQuick also takes longer to detect antibodies after infection compared with fourth-generation and HIV RNA tests, meaning that some early or acute HIV infections could be missed [23]. However, some of these limitations may be unique to the OraQuick test; other rapid HIV tests with better sensitivity and the ability to detect more recent infections could be packaged for home use in the future, addressing some of these problems. Moreover, it may be best to use HBST as a compliment to these other, more precise tests (rather than a replacement), or as a method of engaging those at high-risk who would not otherwise test.

Another key limitation that is common to all existing HBSTs to date is the lack of posttesting follow-up and referrals. Many have argued that one of the most important benefits of clinic-based testing is that test counselors can personally link patients with reactive test results with confirmatory HIV testing and care, or refer those with negative results to other services that reduce HIV risk (eg, testing for other sexually transmitted infections [STIs], preexposure prophylaxis [PrEP], and risk reduction counseling) [24-26]. Modeling studies suggest that because of HBST’s lack of follow-up and referral, widespread use of HBST may actually increase HIV incidence, since individuals who receive reactive results and are not linked with care may delay in seeking it, resulting in onward transmissions during that time [24,25]. Since the OraQuick test became commercially available, OraSure Technologies has maintained a 24-h, toll-free helpline that HBST users can call to receive instructions or guidance about how to conduct the test, posttest counseling, and referrals to HIV care [27]. However, this “passive” approach relies on HBST users to “reach out” for counseling, referrals, and linkage to care themselves, which may be insufficient for many at-risk MSM [10,24]. As of 2015, OraSure estimated that over 500,000 tests had been sold, but of 38,000 calls to their helpline, less than 5% of those were related to posttest counseling needs of HIV diagnosis or treatment [27]. Based on the expected rate of reactive results, these data lend support to concerns that many HBST users may not be connecting to follow-up and referral services after testing, including confirmatory testing or care, STI testing, and additional prevention resources.

Conducting active follow-up with HBST users to provide these services after they test could overcome many of these limitations, while also helping high-risk MSM test more often. Similar to clinic-based testing, active follow-up after HBST could involve having trained paraprofessionals reach out to HBST users to link them with confirmatory testing and medical care, should their results be reactive. This would address one of the key priorities of posttest counseling. Active follow-up could also be beneficial for those with nonreactive results, since counselors could link users with other critical services for reducing their future HIV risk, such as STI testing, risk reduction counseling, safer sex supplies (eg, condoms, lube), PrEP, or postexposure prophylaxis (PEP). However, to be relevant to users, this follow-up contact must be timely, taking place soon after they use a HBST. While it would be difficult to conduct active follow-up with everyone who purchased a test commercially, making brief phone calls to particularly high-risk MSM who agree to receive test kits regularly in the mail might be more feasible. This could be an effective way of reaching a high-risk subset of MSM to encourage them to test more often, while also providing them with the essential posttest services they need (eg, risk reduction counseling, referrals for STI-testing or safe sex supplies, linking those with reactive results with care as soon as possible).

Given this need, we used qualitative research methods to iteratively guide the development and initial evaluation of a “smart” home-based HIV self-testing kit (ie, eTEST) that monitors when kits have been opened in real-time. This system allowed us to actively reach out to users after they use a HBST to provide follow-up counseling and referral over the phone. To inform the development of the eTEST system, phase 1 of this study explored how high-risk MSM would use HBST and their perspectives about receiving counseling and referral after HBST. In phase 2, we then examined the feasibility and acceptability of this system with another small sample of high-risk MSM after using “smart” HBST kits at home.

## Methods

### Participants

A total of 20 participants (n=10 for phase 1, n=10 for phase 2) were recruited via websites and smartphone apps that are commonly used by MSM to meet partners (eg, Grindr, Scruff, and so on) [28]. Eligible participants were (1) 18+ years old, (2) assigned male sex at birth, and (3) fluent in English. They also reported (4) not having been tested for HIV in the last year, (5) having had anal sex without a condom or without having taken PrEP with a casual male partner at least once in the preceding 6 months, and (6) had sex with a casual male partner met on the Web in the past year. Participants were also required to have (7) a stable residence and (8) an iOS or Android (version 4.3 or higher) smartphone with a data plan. The same eligibility criteria applied to participants enrolling in either phases 1 or 2, and participants were required to meet all criteria. All participants reported being male gender, and no participants reported having taken an HBST, PrEP, or PEP in the past.

### Phase 1: Preliminary Interviews

Phase 1 interviews were conducted individually at our offices. The main focus of this phase was to understand HIV testing and how these participants might use HBST, so as to inform our approach to developing the eTEST program, as well as the contexts in which HBST might be used. Key questions posed in this phase included how frequently participants do or think they should test for HIV, their personal barriers to testing, preferences for HBST versus clinic-based testing, views about offering active follow-up after HBST, and preferences about how follow-up might be provided. Whereas the majority of participants in this phase (and phase 2) reported never having tested for HIV before, all had heard about the process and commented on their perceptions based on what they knew about HIV testing. These interviews lasted an hour, were tape recorded (for later transcription and analysis), and participants were paid US $50.

### The eTEST System: Monitoring Approach

After analyzing and interpreting data from phase 1, we used these data to guide the development of the eTEST system. The system used a smartphone app (both iOS and Android versions), together with newly available technologies often used for “Internet-of-Things” (IoT) applications called Bluetooth low energy (BLE) beacons, to monitor when specific users opened a HBST kit that had been sent to them in the mail. BLE beacons are small electronic devices that broadcast a radio signal that can be received by any device equipped with Bluetooth. Whereas BLE beacons are most commonly used to improve smartphone location while indoors (see kontakt.io.Inc., for more information [29]), beacons can also be fit with a variety of sensors (temperature, light, or motion), so that these data can be broadcast to Bluetooth-enabled devices as well.

To set up users in the system, staff entered the user’s information into a secure, staff-side database (Filemaker server). This assigned each user a unique 4-digit personal identification number (PIN), and “pushed” these data to a structured query language (SQL) database that interacted directly with the eTEST mobile app (Figure 1).

**Figure 2:**
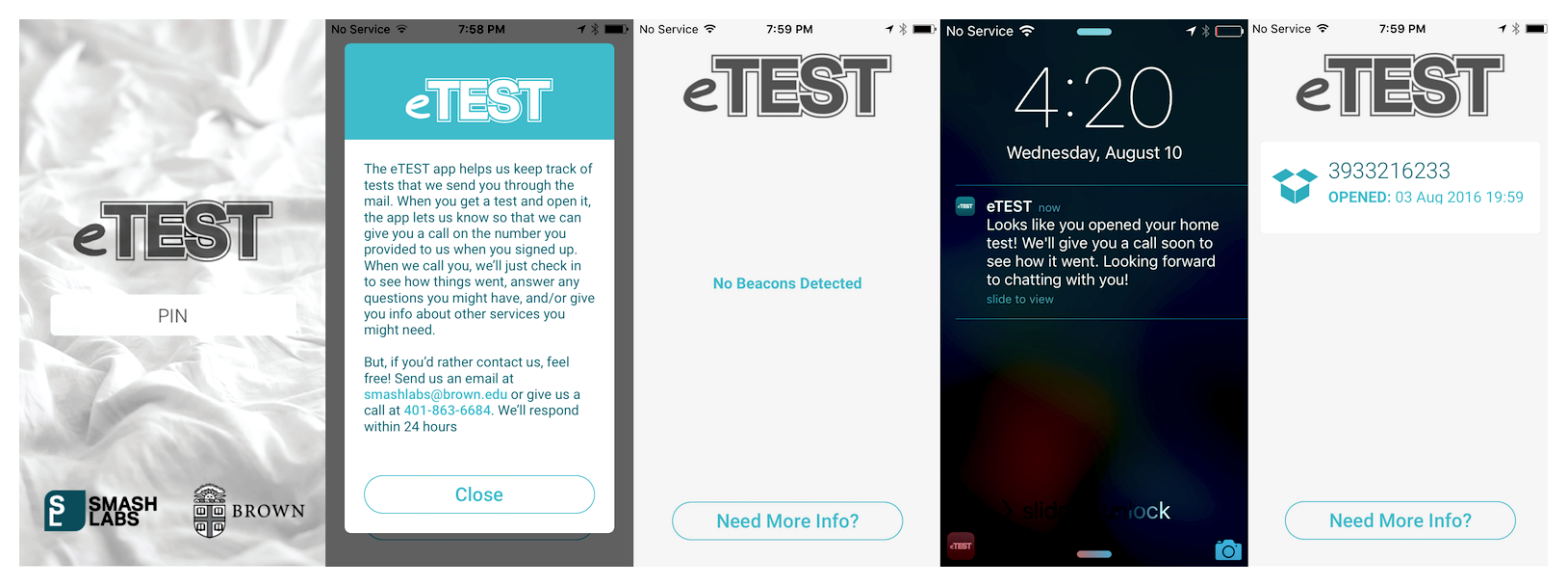
eTEST smartphone app screens.

Staff then guided users through downloading the eTEST app onto their smartphones and entered their assigned 4-digit PIN number in the app, which matched it with the PIN entered through the staff-side database (unmatched PINs produced an error). This way, the app did not collect or store any personal or identifying data, only a participant’s unique study ID (PIN). After successful initialization, the app displayed basic information to the user about the app’s purpose, as well as contact information for study staff (Figure 2).

Users could access this information at any time by pressing a button from the app’s home screen. When preparing an HBST test kit to be mailed, staff fit a BLE beacon to the lid of the plastic test enclosure and logged the serial number of the beacon into a specific user’s record in the staff-side database, which pushed this PIN-serial combination to the SQL database (see Multimedia Appendix 1). To monitor whether a test kit has been opened, the eTEST app used data from the BLE beacon’s ambient light sensor and registered the kit as “open” when the level of ambient light reaching the beacon was >3 lux. As such, when beacons came within range of a user’s smartphone (≈50m) and received sufficient light (> 3 lux), the app pushed a notification to the user and relayed these data to the SQL database, entering a timestamp for the specific PIN-serial combination detected. This identified the user and test kit that was opened in the staff-side database and prompted an automated email to study counselors that notified them of the need to place a call to a user within 24 h of receipt. These notifications were sent only once, when the user first opened the test kit.

We selected Estimote Location beacons for this project, which were released in March of 2016. These beacons required no special programming and only minimal set up to use with the eTEST app. The set up was easily accomplished by staff members and typically took less than a minute (see Multimedia Appendix 2). In addition to the integrated ambient light sensor, these beacons also had a “dark to sleep” feature, that tells the beacon to stop broadcasting data until it receives a level of light that is above a customizable threshold. Using this feature, beacons can be effectively “muted” until the beacon receives sufficient light (>3 lux). This capability was important for our use case, since our goal in designing the eTEST system was to ensure that it could detect HBST use over long periods of time without requiring any intervention from users (other than initially downloading the app). That is, we wanted to design the eTEST system to successfully capture HBST kit openings, even when users had the app running in the background, when they had “killed” the app, or when the phone was in sleep mode. Accomplishing this proved to be a somewhat difficult technological hurdle in iOS. With a typical BLE beacon that continuously broadcasts data, apps that have been “killed” can be “woken up” when a beacon comes within range (typically ≈50m) of the phone. However, iOS kills these processes again after a few minutes. This posed a problem for our use case, since the beacon (enclosed in a test kit) would often be expected to come within range of a user’s phone long before they actually open the test kit. That is, the time between users receiving a test kit and when they actually open it will often be significant. So, running the app’s processes briefly only after users receive the test posed a high risk for missing opening events, if iPhone users had killed the eTEST app or if the phone was in sleep mode. Using the “dark to sleep” feature of the Estimote Location beacons, we directed the beacons to begin broadcasting only after it received a sufficient amount of light, prompting the app to briefly “wake up” to sync with the server. Preliminary testing in the lab showed that, using this feature, test kit openings were indeed successfully detected in virtually all iPhone states (eg, app “killed,” phone in sleep mode), so long as the app had been downloaded and had been successfully paired with a 4-digit PIN and beacon serial number. Of note, this limitation is not relevant to Android users, since a process can be run in the background at any time.

### Phase 2: Usability Testing and Follow-Up Interviews

After initially developing the eTEST system, we then iteratively built several app releases and tested them with various devices and states, both in the lab and during a number of “hallway tests” [30]. Once a working version was constructed, we moved on to phase 2, which involved conducting a usability test with target users [31]. In this phase, our goal was to preliminarily test how well the eTEST monitoring and follow-up system worked in a real-world scenario, where participants were using their phones, the app, and the HBST kits under typical circumstances. We explored several questions that could affect how well the system works, including whether users opened the “smart” HBST kit within range of their smartphones, whether they actually used the test soon after initially opening it, whether the light threshold (>3 lux) was sufficient to detect openings in dimly-lit environments, and whether detection was successful across operating systems (ie, iOS and Android) and phone or app states (eg, app “killed” or phone in sleep mode). We also examined ease of use, perceived utility of (and comfort with) receiving follow-up calls, the length and timing of calls, and concerns about confidentiality or privacy.

To address these questions, participants in phase 2 first met with research staff in-person to learn about the study, downloaded the eTEST app onto their smartphones, and were provided with a BLE-enabled OraQuick home HIV test. They were asked to use the test at some point within 7 days, but were provided with no other instructions about how to use the app, other than to keep it downloaded onto their device during that time. This allowed us to explore how well the eTEST system worked in a variety of phone states. After they opened the test, a qualified HIV test counselor (QHTC) followed up with participants over the phone within 24 h. During these calls, QHTCs provided a “typical” sequence of posttest counseling, including: (1) discussion of the test, its use, and results; (2) offering counseling to reduce HIV risk behaviors; and (3) offering referrals for STI testing, safe sex information and supplies, PrEP consultation, and other medical care or counseling (mental health, alcohol or drug). Afterward, counselors conducted a brief follow-up qualitative interview about their experience and opinions of eTEST. These interviews lasted about 30 min, were recorded, and participants were compensated $50. All procedures were approved by the Brown University Institutional Review Board.

### Analysis

All interviews and counseling calls were transcribed, and transcripts were reviewed and coded manually by study staff. Content and codes were then analyzed thematically. Regular discussions helped to arrive at themes that emerged across participants, and specific quotes were excerpted from transcripts to illustrate these themes.

## Results

### Phase 1: Preliminary Interviews

Ten participants completed the phase 1 interview. Only 3 of these participants (30%, 3/10) reported having tested for HIV in their lifetime, and of those, all reported having tested only at a clinic or outreach event (see Table 1).

However, all participants thought that MSM in general should be tested more often than they tested themselves: whereas no interviewees reported having been tested in the past year (since this was a criteria for eligibility), most suggested that MSM should be tested at least every six months, and with more frequent testing for those who engaged in riskier sexual behavior. Those that had tested before noted that they appreciated testing in person with a paraprofessional because the counselors were often understanding, skilled, and could make testers feel comfortable. They also noted that in-person HIV tests were also relatively quick and easy to do. However, most participants noted drawbacks of in-person HIV testing, with most identifying nervousness as a key downside:

Basically throughout the entire duration of the tests or going into the clinic up to getting your results, I felt nervous about what the results might be, even though I felt for my case that it was extremely unlikely that something would come back positive.

Participant 1, 30-year-old white male, last tested 3.2 years ago

Despite the professionalism of clinic staff, there were also concerns about the confidentiality of clinic-based testing:

The confidentiality. That’s a big thing (...) because I know people who work in there—I was afraid of like the rumor getting around.

Participant 2, 21-year-old Hispanic male, last tested 1.1 years ago

#### Barriers to More Frequent Testing

Most participants noted that the most important barrier was that they perceived themselves to be at low risk, despite meeting the study inclusion criteria. However, another key theme mentioned by most participants was the inconvenience involved:

It’s something that I actually have to go out and do, and it just kind of slips my mind a lot, more than anything. And then it’s like I know I should, and then I don’t know, I’m really good at procrastinating.

Participant 3, 36-year-old white male, last tested 2.1 years ago

This concept illustrates the importance of making testing as quick and easy to do as possible. By delivering free HBST kits directly to a subset of high-risk individuals at recommended intervals (eg, at least once every six months), the convenience of testing at home may help overcome other important barriers like low risk perceptions.

#### Pros and Cons of Home-Based Self-Testing (HBST) and Preferences for HBST Versus Clinic-Based Testing

Most phase 1 participants noted the convenience of HBST as its biggest strength:

A doctor is not always going to be in the office when you call. Sometimes it’s hard to reach a doctor. It could take weeks. But somebody (who) buys this doesn’t have to wait that long at all. The same day they buy it, they can get results the same day.

Participant 4, 42-year-old Hispanic male, never tested

Most participants also noted that HBST could be more confidential and private than clinic-based testing:

It’s extremely private...nobody will know the results but you. It’s a good way to be more private or at ease for certain people.

Participant 1, 30-year-old white male, last tested 3.2 years ago

Finally, several participants also mentioned that doing the HIV test themselves might make them feel more empowered and proud of having taken responsibility for their own health:

I think it gives the individual more power and control over the situation. In the past, you had to go to a doctor in order to get tested. But now, the individual has the power to access that knowledge.

Participant 3, 36-year-old white male, last tested 2.1 years ago

**Table 1:** Participant demographic characteristics.

Characteristics		Median (IQR^a^) or n (%)	
		Phase 1 (n=10)	Phase 2 (n=10)
Age (years), range: 21-67, mean (SD)		29 (18)	30 (31)
**Race**			
	White	8 (80)	9 (90)
	Black or African American	2 (20)	1 (10)
	Ethnicity (Hispanic or Latino)	2 (20)	0 (0)
**Relationship status**			
	Single or never married	8 (80)	7 (70)
	In a committed relationship	0 (0)	0 (0)
	In a domestic partnership	0 (0)	0 (0)
	Married	0 (0)	1 (10)
	Separated	2 (20)	0 (0)
	Divorced	0 (0)	1 (10)
	Widowed	0 (0)	1 (10)
**Education**			
	High school diploma or general educational development	1 (10)	1 (10)
	Some college education	6 (60)	4 (40)
	College graduate	2 (20)	3 (30)
	Graduate or professional degree	1 (10)	2 (20)
**Income**			
	$0-$29,999	5 (50)	3 (30)
	$30,000-$99,999	5 (50)	6 (60)
	$100,000 or more	0 (0)	1 (10)
**Sexual identity**			
	Gay	9 (90)	8 (80)
	Bisexual	0 (0)	1 (10)
	Other	1 (10)	1 (10)
Never tested for HIV		7 (70)	9 (90)
Average years since last HIV test^b^, mean (SD)		2 (2.1)	16 (--)

^a^IQR: interquartile range

^b^Among participants reporting having tested for HIV in their lifetimes.

In addition to these strengths, however, interviewees also noted a number of important drawbacks of HBST compared with more traditional, clinic-based testing. Many target users noted a fear of conducting the test incorrectly when doing it on their own as a key reservation:

I would be super terrified that I would be messing something up, and I wouldn’t actually know how to use it, so I don’t know if there’s a way to somehow make a YouTube video or something or have (...) like a Q-R code that went to a video that showed how it’s done. As a millennial, I don’t like to read. Things need to be like quick and easy.

Participant 5, 21-year-old white male, never tested

Participants also noted concerns about the potential consequences of getting results from an HIV test while alone as another drawback of HBST:

What happens when the result comes back positive? With a clinic or some other kind of professional present, there’s someone who can handle the situation in some ways if I’m not able to myself. And here, someone who is not able to handle the situation, for example by calling a professional or going to the doctor afterwards, would be left alone.

Participant 3, 36-year-old white male, last tested 2.1 years ago

Finally, when asked whether they would prefer using a HBST kit versus testing at a clinic, participants overwhelmingly said they would prefer to use HBST:

You can’t say ‘Oh, I don’t want to go make that doctor’s appointment when you’ve got this sitting in your cabinet and you can use it. (...) I can’t think of too many cases where you wouldn’t just want to do (HBST) to save yourself the time, hassle, and stigma (...) of going to the clinic.

Participant 8, 48-year-old African American male, never tested

#### Perceptions of Offering Follow-Up after HBST

When we asked these participants about their views of offering more active follow-up counseling and referrals after HBST by reaching out to users (as opposed to providing users with the phone number of a 24-h helpline), most thought this would be helpful. The advantage most commonly identified by participants was that actively following up with HBST users would show concern and provide support:

There’s something always great about talking to another human being. (...) You’re just left kind of very vulnerable out there, and you don’t have the structure around you that you would if you went to a medical clinic, and that sense of direction and extra support from people to come in and help you would be nice. It’s bringing in that support structure that’s already in the medical clinic into the home.

Participant 2, 21-year-old Hispanic male, last tested 1.1 years ago

Participants also suggested that this active follow-up could be especially important for users who receive reactive results through a HBST kit, specifically because it could help link them with confirmatory testing and follow-up care:

I suppose like if you did find your result to be positive that could be kind of shocking and that you might really not be sure what to do. It would be an advantage to have someone there with you to talk about what to do.

Participant 3, 36-year-old white male, last tested 2.1 years ago

Another commonly identified advantage was that having a counselor reach out could encourage users to follow up with referrals and test again in the future:

I think the reaching out, it shows concern. I think it would spark enthusiasm to get serious about (getting tested).

Participant 7, 42-year-old African American male, never tested

A few participants thought offering active follow-up was either unnecessary because users could find information by themselves, or because it could deter some users from testing who would prefer not to speak with someone afterward:

I might want additional information or referrals, and assuming the test doesn’t offer it, then I would be able to get that. But one of the biggest advantages of this test that I see is what people who are afraid of talking to a doctor or a person about HIV could use it. And I would rather have those people be able to use the test than not take it at all.

Participant 9, 24-year-old white male, never tested

Finally, participants overwhelmingly believed that receiving a phone call would be the best way for counselors to follow up after using an HBST kit. However, others suggested they would be equally comfortable with text-based forms of communication, like chat, text, or even email:

I think (calling is) very old, outdated—people have changed, technology has changed. For me, I guess I respond best by email or texting. Maybe if there was an app, that would be super cool.

Participant 5, 21-year-old white male, never tested

### Phase 2: Usability Testing

Ten participants completed phase 2, and of these, only 1 (10%, 1/10) reported having ever tested for HIV. No participants reported having taken or heard of HBST prior to participating. Seven participants used iOS smartphones and 3 used Android smartphones. All users in phase 2 reported having tested negative.

Nine of ten opening events were successfully captured, and by participant report, notifications typically registered within a minute after having opened their HBST kits. Follow-up calls were successfully placed within 24 h to each of the 9 users for whom the system worked as expected. Follow-up interview data with the remaining user suggested that the system failed because his smartphone was being repaired when he opened the test. Together, the results of our lab testing and this usability test show that the eTEST system was able to successfully detect when “smart” HBST kits were opened by users, except in less common circumstances in which participants’ phones were not functional.

Phase 2 qualitative interviews also suggested that our strategy of monitoring when users opened their tests using a BLE beacon and smartphone app was a good fit with how participants used their tests at home. For example, one key question about this approach was whether users actually took their home-based tests soon after initially opening the test enclosure, or whether there would be a delay, making efforts to follow up after initially opening the test less relevant. However, results suggest that all users took their tests within a few minutes after opening them. Since the smartphone can only detect data from beacons within a certain proximity (≈50 m), another question was about whether users would have their phones nearby them when they took the test. All participants reported having their phones within a few feet of the test when opening it, with some adding that they used other features of their phones to help them with the test (eg, the “timer” app). Finally, users also noted that the eTEST app did not appear to drain their phone battery noticeably, and that it used very minimal data.

### User Perspectives

Feedback from these participants about home-based testing and the eTEST system was very positive. Participants emphasized the simplicity of the home-based test itself, with several also noting that providing the test kits in the mail at regular intervals may encourage them to test more regularly:

It was just so easy to follow the directions (of the HBST). They seemed to think of everything. I kind of enjoyed doing it at home, it seemed very straight-forward. If that thing came in the mail on a schedule, I think somebody would be much more likely to do it, as opposed to like ‘Oh, I’ve got to go down there) to get tested).

Participant 17, 22-year-old white male, never tested

Many participants also noted that having QHTCs follow up with them after they tested over the phone could provide important support, and may help many overcome obstacles they have in seeking help afterward:

I find it unnerving in these kinds of circumstances to take the first step of getting that information, so to have someone else there saying ‘Oh, I have this information for you right now,’ I think is very helpful, because sometimes (finding) those things on your own can be a bit stressful.

Participant 11, 22-year-old white male, never tested

Several also noted that following up with users after they test at home could be especially important for those who have reactive results (hypothetically), and could play a role in motivating these users to seek timely confirmatory testing and follow-up care:

I think it’s good to have a follow-up, in case there was a situation where it was positive, I would know that someone would be contacting me to give me clinical information and places I could call to set up an appointment or get in contact to continue the process of getting a diagnosis. It definitely would force you to continue with a follow-up.

Participant 13, 27-year-old white male, never tested

Finally, some users said that, even for those who test negative, follow-up calls could be helpful for connecting users with referrals for other services (STI testing, in particular):

Most of the advertising you see is for HIV (testing), so I’d never heard of a place or a clinic you could go to have (STI testing) done until now.

Participant 11, 22-year-old white male, never tested

Participants also provided feedback about the method of offering counseling and follow-up after testing (ie, phone calls), as well as the timing and duration of the calls (which lasted on average 10 min; range 5-19 min). Most participants preferred to receive counseling and follow-up over the phone, but a few noted having other options to seek information and ask questions might be a better fit for some users:

The phone, I imagine, would be the simplest for a lot of follow-up conversations. The questions back and forth are easier to answer than maybe like an email would be. With the way younger people are used to technology these days, though, it’s more about text messaging and something that’s not as personal as actually seeing somebody.

Participant 14, 62-year-old white male, never tested

All participants agreed that the duration of the calls was appropriate, and that the topics addressed during the follow-up were relevant and useful. However, they disagreed about the timing of calls. Nearly all suggested that placing these calls within 24 h would ensure that the conversation would be most relevant; however, some participants said they would have preferred getting the call even sooner. Several noted that the best time to call might depend on the test results, and that, if they had received reactive results, they may want to be contacted sooner:

(If the results were reactive,) my gut says within an hour, I would want the phone to ring. If it was too long, who knows what I might do? It’s so treatable that I highly doubt (anything would happen), but I think I would still want the call.

Participant 20, 45-year-old white male, never tested

## Discussion

### Principal Findings

Our goal in developing the eTEST system was to make testing for HIV more accessible, especially for high-risk MSM who encounter barriers to clinic-based testing services. HBSTs for HIV could be a key opportunity to help achieve this, since they eliminate some of the most common barriers that prevent high-risk individuals from testing more regularly in clinics [10,16,32]. One way to encourage more regular testing might be to deliver HBST to those at highest risk at specific intervals (eg, 3 months or 6 months), so that new infections can be detected earlier [24,25]. However, to ensure that those who use HBST can be efficiently connected with vital posttesting resources (eg, confirmatory testing and care), a tool that can monitor HBST use in real time and facilitate post-test follow-up and referral is needed. Results from this study provide strong support for the feasibility and acceptability of a tool we developed to accomplish this.

Consistent with past research [11,12,32], our preliminary interviews with high-risk MSM who met partners on the Web and who had not tested for HIV in the last year suggest that access to HBST may indeed encourage these individuals to test more regularly, and that most viewed HBST as more convenient, confidential, private, and empowering. Nearly all participants in both phases also reported that they believed they would be more likely to test using HBST than getting tested at a clinic. However, another important goal of developing the eTEST system was to address one of the key limitations of HBST: The lack of follow-up and linkage to care after testing at home. The results of our initial interviews suggested that, whereas some high-risk MSM may not prefer it, most saw advantages of offering counseling and referral over the phone after using a HBST. Specifically, participants noted that phone calls from an HIV test counselor would show concern, provide reassurance, and could connect them with professional help. Participants who actually experienced this sequence of follow-up shared these perspectives. Many also discussed ways to reduce their risk with counselors during the calls and received referrals for STI testing and PrEP.

Usability study findings provide key preliminary support for the feasibility and acceptability of our approach to monitoring participants’ use of HBST kits (thereby enabling timely, active follow-up phone calls). In developing the system, we hoped to ensure that it would work without requiring users to interact with their phones, since we expected that most would-be users of this system will not keep the app running or have their phones actively awake or in-use at the time they test. This is particularly important, given that we designed this system to be used over long periods of time, since even high-risk MSM would be encouraged to test once every 3 months, at most. With our approach, we were able to successfully detect 90% (n=9) of test kit opening events across both Android and iOS smartphones, with the one “missed” event occurring due to the user having his phone repaired. Also key to long-term acceptability was ensuring that the eTEST app was not overly burdensome and did not interfere with users’ day-to-day use of their phones. All usability study participants kept the eTEST app on their phones for the week required and reported that the app did not noticeably affect their phone’s battery, data usage, or memory (iOS: 33.4 MB, Android: 20.0 MB). Similarly, usability testing also confirmed that our strategy for monitoring HBST kits addressed many of the parameters needed for the system to work successfully. Specifically, we found that users took their tests soon after opening the test’s plastic enclosure, suggesting that monitoring when the test kit is opened serves as an appropriate marker of when they actually took the test. Results also confirmed that participants often had their phones very close to them when they completed the test, with some using other features of their phones to help them with the test (eg, to time the required 20-min wait before reading results). This was important to confirm, since users’ phones must be within approximately 50 m of the beacons in the test kits to capture data being transmitted from them.

Perspectives from usability study participants also provided support for the utility, duration, and timing of follow-up counseling and referral phone calls. Echoing phase 1 results, several usability study participants reported that receiving the follow-up calls provided them with support and reassurance, even though all test results were negative. Many also noted that the counselor’s ability to provide them with further information about HIV, the test, and other prevention services was a strength. Finally, usability participants also noted that follow-up calls were timely and brief. Some reported that it might be ideal to connect with users who receive reactive results sooner than 24 h later; however, most said that the timing seemed appropriate and that the duration was brief enough to encourage them to take the call. Finally, participants also noted that they had no concerns about the privacy of the app or its features (eg, push notifications), since all register within a few minutes of opening their tests, ensuring that they were already in a private location when it displayed. Additionally, most had already made use of their devices’ lock screen feature. Overall, these perspectives suggest that the HBST kits were easy to use and that the follow-up phone calls could be an important way to offer support to HBST users in general. They also suggest that reaching out to users afterward could be an important way to link them with other important resources like STI testing and PrEP, and with confirmatory testing and care (for those who receive reactive results).

### Limitations

Several important limitations should be noted. First, as this system relies on a smartphone app that uses data, it can only be used by those with iOS or Android (version 4.3 or higher) smartphones that have a data plan. Market research suggests that up to 68% of the adult population in the United States currently own smartphones [31], and this figure is likely to grow exponentially in the future. iOS and Android users comprise 95.3% of these smartphones [32]. As demonstrated in our usability study, the eTEST system could be used to target MSM who use smartphone apps to meet partners on the Web, and therefore already use smartphones. However, some of those at highest risk for HIV may not own or use smartphones [33,34]. Similarly, the eTEST system was designed with the future goal of facilitating a free HBST program that involves regularly sending kits to participants in the mail. As such, its utility among particularly vulnerable and at-risk individuals, such as the homeless, may be limited. However, future research could explore alternative methods of test delivery and detection that are more fitting for individuals with unstable housing. Second, several characteristics of the technologies used may lead to “missed” test opens. Opening events are successfully captured even when the app is not running (even in the background) and the phone is in sleep mode; however, the phone must be turned on and the app must be on the phone and registered (ie, successfully paired with a 4-digit PIN number) at the time the test is opened. In addition, the phone must be within about 50 meters of the beacon. Finally, it should be noted that, while we are excited about the implications this work may have for improving the delivery of HBST, we do not believe HBST should replace testing at a clinic. More research is needed, but we believe the most fitting approach might involve encouraging HBST use in between visits to a clinic to get tested.

### Future Research

Whereas these data show that the system operates as intended and that the perspectives and preferences of a small pool of intended users (ie, high-risk MSM) are supportive, additional research is needed to address a number of key questions about the utility of offering HBST programs to high-risk groups. Specifically, a key next step should involve testing whether HBST-based strategies improve rates and frequencies of HIV testing among high-risk populations that typically test infrequently, and in particular, whether these strategies are capable of reducing incidence among them as a result. To address this, future research should explore whether HBST approaches detect more new infections and facilitate earlier diagnoses compared with relying on clinic-based testing alone, as well as whether the eTEST approach is successful in linking users with key additional services that can prevent or reduce the risk of future infections (eg, confirmatory testing and care, STI testing, PrEP). With these data, researchers can model the potential population-level effects of incorporating HBST-based strategies into community testing programs on HIV incidence. Arguably the most important benefit of conducting posttest follow-up after HBST is the potential it has for linking those who receive reactive results with confirmatory testing and care as soon as possible. This could suggest that it is best to focus follow-up efforts toward these users in some way; for example, by using the eTEST system to generate an automated SMS text message asking participants to report the results of their test, and then placing follow-up calls to only those who report receiving a reactive result. However, the lack of follow-up and referral to other prevention services (eg, STI testing, PrEP) for those who test negative has also been cited as another key limitation of HBST [24,25]. Linking users with these services may also provide valuable benefits by reducing future risk. As such, we believe that the question of “how” to offer follow-up and “with which users” is a question best left to future studies. For this reason, this manuscript details the rationale and approach for offering follow-up that could be useful for both types of users.

In summary, this study illustrates our use of emerging IoT technologies to enable more robust delivery of counseling and referral services alongside HBST for HIV. Iterative, qualitative research provided preliminary support for the feasibility and acceptability of using this approach to monitor when HBST users take their tests in order to facilitate timely follow-up phone calls from counselors. We are excited about the potential these technologies have for encouraging high-risk individuals to test more frequently; however, further work is needed to explore acceptability among target users over broader time periods (eg, months and years) as well as the effects that frequent HBST and follow-up phone contact have on rates of HIV testing and linkage to other prevention resources.

## Appendix

Multimedia Appendix 1 The OraQuick home-based self-test for HIV, fit with Estimote Location beacon.

Multimedia Appendix 2 The Estimote Location Bluetooth low energy (BLE) beacon.

## Abbreviations

BLE: Bluetooth low energy
HIV: human immunodeficiency virus
HBST: home-based self-testing
IoT: Internet-of-Things
MSM: men who have sex with men
