# Supplementary figures and images for: eTEST: Developing a Smart Home HIV Testing Kit that Enables Active, Real-Time Follow-Up and Referral After Testing
Source: JMIR Mhealth Uhealth. 2017 May 8;5(5):e62. doi: 10.2196/mhealth.6491 (PMC5440737; doi:10.2196/mhealth.6491)

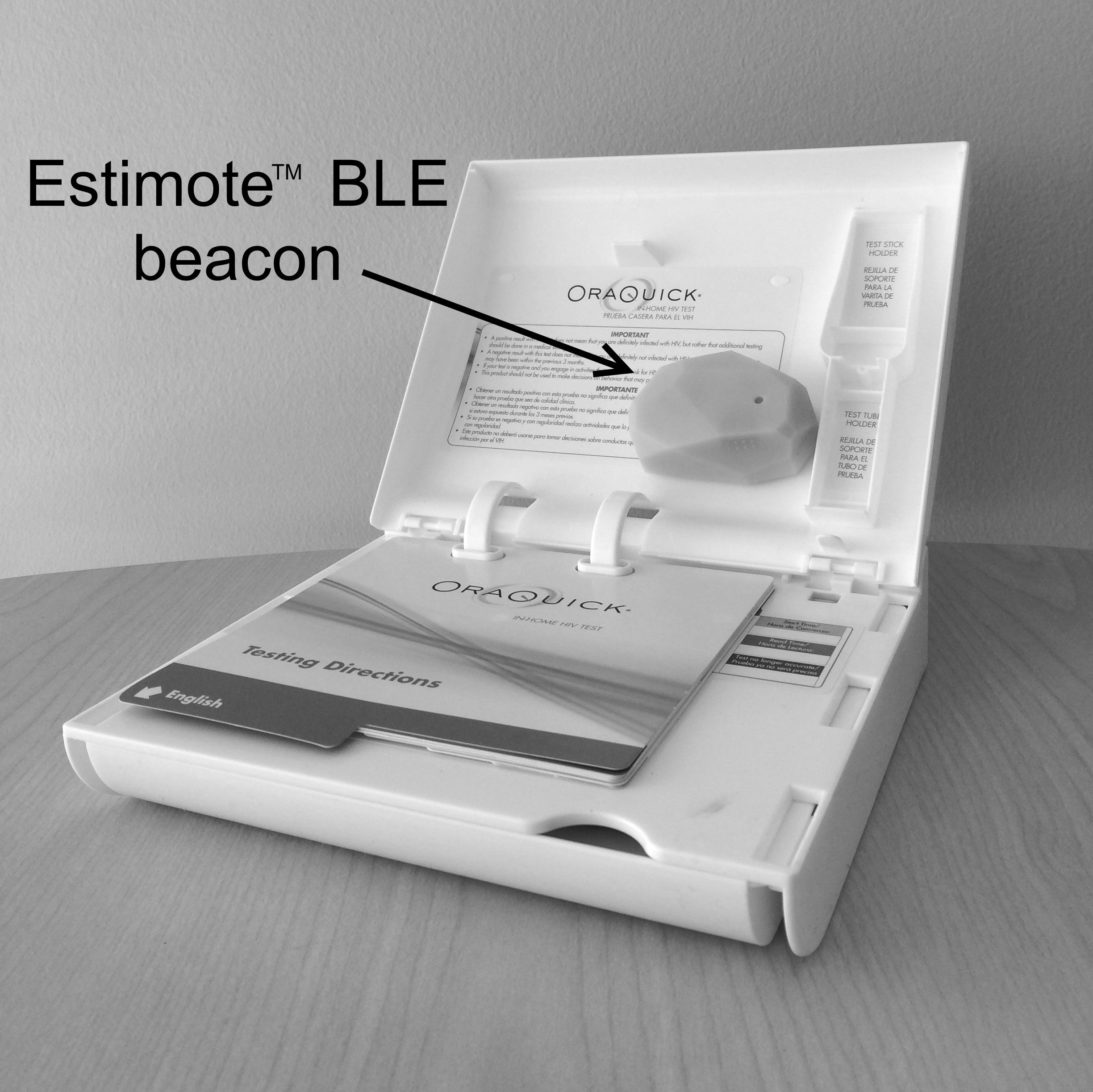

Supplement: Multimedia Appendix 1 [file mhealth_v5i5e62_app1.jpg]

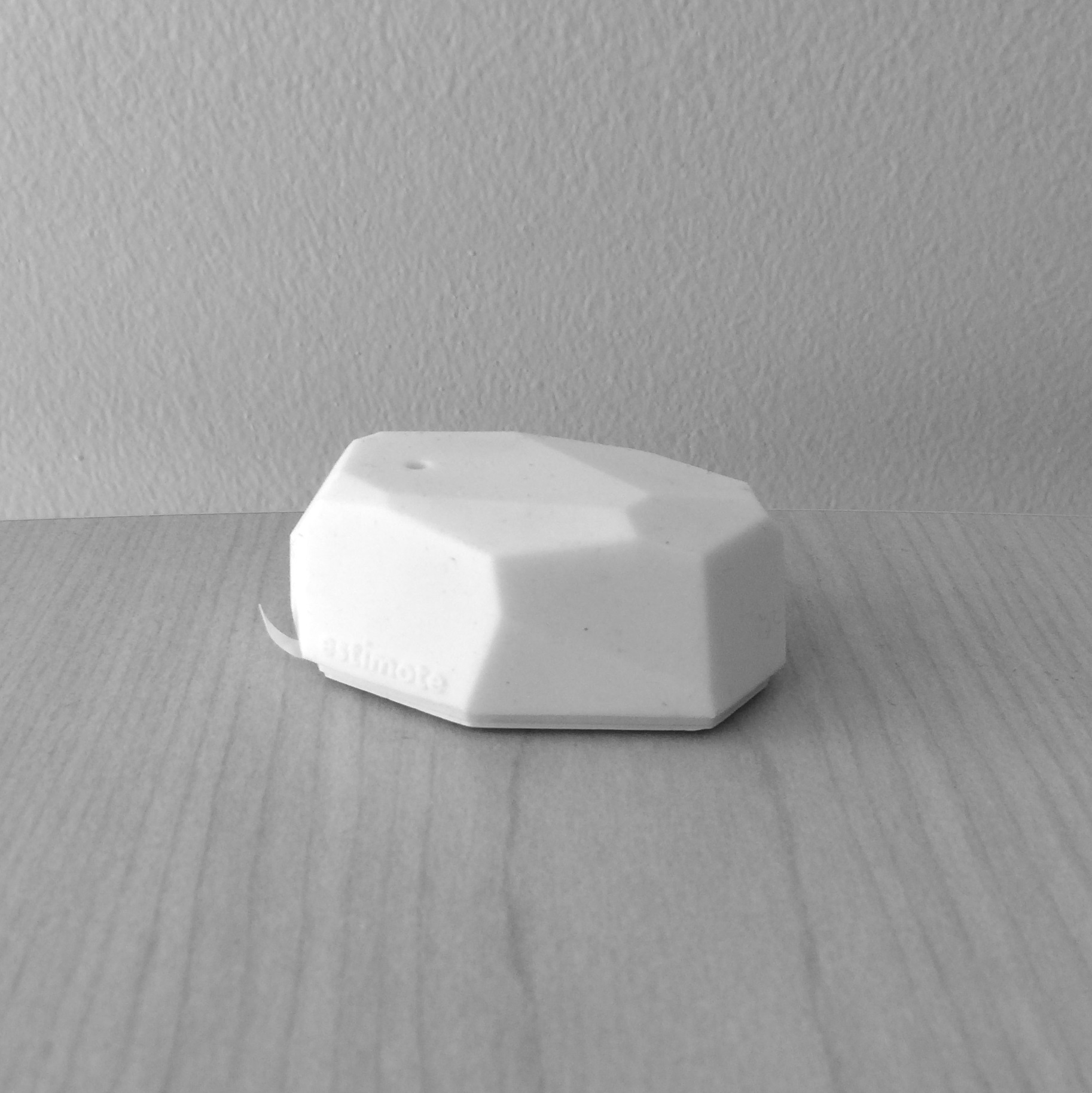

Supplement: Multimedia Appendix 2 [file mhealth_v5i5e62_app2.jpg]
